# Diffusion Nitride Coatings for Heat-Resistant Steels

**DOI:** 10.3390/ma16216877

**Published:** 2023-10-26

**Authors:** Khrystyna Berladir, Tetiana Hovorun, Vitalii Ivanov, Djordje Vukelic, Ivan Pavlenko

**Affiliations:** 1 Department of Applied Materials Science and Technology of Structural Materials, Faculty of Technical Systems and Energy Efficient Technologies, Sumy State University, 2, Rymskogo-Korsakova St., 40007 Sumy, Ukraine; kr.berladir@pmtkm.sumdu.edu.ua (K.B.); hovorun@pmtkm.sumdu.edu.ua (T.H.); 2 Department of Automobile and Manufacturing Technologies, Technical University of Kosice, 1, Bayerova St., 080 01 Presov, Slovakia; 3 Department of Manufacturing Engineering, Machines and Tools, Faculty of Technical Systems and Energy Efficient Technologies, Sumy State University, 2, Rymskogo-Korsakova St., 40007 Sumy, Ukraine; 4 Department of Production Engineering, Faculty of Technical Sciences, University of Novi Sad, Trg Dositeja Obradovica, 6, 21000 Novi Sad, Serbia; vukelic@uns.ac.rs; 5 Department of Computational Mechanics Named after Volodymyr Martsynkovskyy, Faculty of Technical Systems and Energy Efficient Technologies, Sumy State University, 2, Rymskogo-Korsakova St., 40007 Sumy, Ukraine; i.pavlenko@cm.sumdu.edu.ua; 6 Department of Industrial Engineering and Informatics, Technical University of Kosice, 1, Bayerova St., 080 01 Presov, Slovakia

**Keywords:** process innovation, industrial growth, heat-resistant steel, AISI A290C1M, ion nitriding, nitriding in powder mixture, melamine, microhardness, wear resistance

## Abstract

The effect of ion nitriding and nitriding in a melamine-based powder mixture on the structure and properties of AISI A290C1M steel was studied in the paper. Using ion nitriding made it possible to shorten the technological cycle’s duration by 5–6 times compared to two-stage nitriding, optimize the diffusion layer’s composition, provide a technologically simple process automation scheme, and improve the quality of nitride coatings. After the proposed mode of ion nitriding, a saturated layer depth of 0.25–0.32 mm, hardness up to 1000 HV, and an increase in wear resistance by 2.17 times were obtained. Using 95% melamine + 5% sodium fluoride during nitriding in a powder mixture significantly simplified the technological process. It did not require additional expensive equipment, which in turn significantly simplified the nitriding process with energy savings. The proposed technology and the composition of the mixture contributed to a significant acceleration of the nitriding process of AISI A290C1M steel, compared to traditional gas nitriding, and to obtain a hardness of the nitride layer of 970 HV and an increase in wear resistance by 2.6 times. A nitriding speed is explained by a significantly higher amount of atomic nitrogen when using melamine instead of ammonia and by the almost simultaneous disintegration of nanodispersed particles when the nitriding temperature was reached. After nitriding in a powder mixture, steel was subject to the slightest wear.

## 1. Introduction

Increasing the reliability and durability of machine parts and structures, improving their quality and efficiency of work, saving metals, and fighting against corrosion and wear are the priority tasks of science and technology. Solving these problems is primarily related to the need to develop and implement highly effective methods of increasing the structural strength of metal materials. In this regard, methods of surface strengthening of parts [1], among which the most promising and widespread are methods of chemical and thermal treatment [2], play an essential role in solving this problem. Therefore, the development of new and the improvement of existing types and technological processes of chemical-thermal treatment [3], which change the structure and phase composition of the surface layer of the part, are relevant. They will allow optimal properties and characteristics of the product to be obtained [4].

Chemical-thermal treatment consists of changing the chemical composition and structure of the surface layers of parts by their diffusion saturation at elevated temperatures with one or more elements [2]. Depending on the saturating element, the following types of surface saturation are distinguished: single-component: cementation—carbon saturation [5]; nitriding—nitrogen saturation [6]; multi-component: nitrocarburizing—saturation with nitrogen and carbon [7]; carbonitriding—saturation with carbon and nitrogen [4].

The authors [8] investigated the change in the microstructure and properties of ASSAB 760 steel during the process of cementation and quenching. It proved the effectiveness of the formation of the martensite and austenite microstructures.

The effect of heat treatment on modified Hadfield steel’s microstructure and wear resistance was investigated in [9]. It was shown that increasing the heat treatment temperature and decreasing the heating rate positively affect the dissolution of secondary carbides and transformation martensite, significantly improving the hardness, wear resistance, and friction coefficient of Cr + Ni-modified Hadfield steel.

High mechanical and thermal loads characterize the operating conditions of piston compressor rods. Mechanical loads from gas pressure act on the piston. The rod works under conditions of constant or periodic friction with high thermomechanical loads and is subjected to various types of compressive, bending, and torsional stresses. The material for manufacturing the piston compressor rod must have high strength and hardness on the surface and a viscous and plastic core. The main reason for the rod’s destruction is the surface’s wear [10], which requires additional strengthening technologies.

Nowadays, many technological processes of nitriding have been developed. Nitriding processes are classified according to [11]:

- Composition of the medium—nitriding in gases [12], liquids [13], and solid media (in pastes or powders) [14];
- Process temperature—low-temperature [15], medium-temperature [16], high-temperature [17];
- Pressure in the reactor—at increased, decreased, pulsating pressure;
- Method of supplying energy—heating due to convection, radiation, low-temperature plasma, induction electronic and laser heating [18];
- Type of furnace equipment—chamber, shaft, crucible, and other furnaces.

The method of nitriding in dissociated ammonia using furnace heating, which is widely used in industry, has such disadvantages as the long duration of the process, the difficulty of nitrogen saturation of steels, and the formation of a brittle ε-phase on the surface of the part.

The process of ion nitriding allows not only the disadvantages listed above to be removed, but also the following advantages: the possibility of adjusting the processing parameters in a wide range of modes and, due to this, the structure, phase composition, hardness, wear resistance; high saturation speed; greater efficiency of the process; is non-toxic and meets the requirements for environmental protection [19,20]. But to carry out ion nitriding, it is necessary to use high-energy vacuum installations [21]. Based on the above, the task of continuously improving existing and known technologies arises.

For example, in paper [22], the authors conducted a series of complex studies for tool steels AISI H13, AISI P20, and N-8550 to establish the best-acquired properties of the surface layer after solid, gas, and plasma nitriding. As a result, the hardness and wear resistance of the surface was increased for all samples. However, the authors proved that using hard nitriding for tool steels shows the highest wear resistance values compared to gas and plasma nitriding.

Dobrocky et al. [23] compared the effects of gas and plasma nitriding for 42CrMo4 alloy steel on its surface roughness and dimensional accuracy. The analysis of the results of the experiments confirmed the expected increase in surface hardness of the material, reduction in the coefficient of friction, and increase in wear resistance. However, the controlled parameters of the surface roughness and degrees of accuracy of the dimensions of the samples were found to deteriorate, more so when using gas nitriding.

The advantages of plasma nitriding and the prospects for its application are described in [24] for heat-resistant, relaxation-resistant steel 38CrMoAl and in [25] for cold-working steel SKD11.

Fraczek et al. [26] studied the process of ion nitriding on 316L austenitic steel, and two experiments were conducted on the placement of nitride elements in the glow discharge chamber. The authors analyzed the influence of the process parameters on the nitrogen diffusion depth and proposed a new mechanism of ionizing nitriding with the active screen method.

Therefore, the purpose of the paper is the rational choice of chemical and thermal treatment of the AISI A290C1M steel, during the development and research of a diffusion nitride coating to ensure the strength characteristics of the part using methods of ion nitriding and nitriding in a powder mixture based on melamine.

The following tasks were set and solved in the paper to achieve the goal mentioned above:

- Determine the advantages and disadvantages of gas nitriding in comparison with ionic nitriding and nitriding in a mixture of melamine and sodium fluoride;
- Choose such a saturating composition that will significantly accelerate nitriding processes, compared to gas; develop technologies that can be implemented without special equipment;
- Choose the necessary parameters of chemical and thermal treatment: ranges of technological parameters of nitriding (temperature, composition of saturating media, duration of saturation), which allow purposefully varying the phase composition of the hardened layer over a wide range.

## 2. Materials and Methods

### 2.1. Materials

The following requirements are imposed on the materials used to manufacture piston compressor rods: high mechanical strength, wear resistance, high corrosion resistance, good resistance to temperature and gas pressure, sufficient heat resistance, sufficient viscosity and hardness, and high endurance limit. Surface wear is the main reason for the destruction of such a part.

The main requirements for the material are high strength with high hardness of the friction surfaces, which can be achieved by obtaining a nitride layer with a hardness of HV 1000–1200. Therefore, heat-resistant, relaxation-resistant steel AISI A290C1M (DIN 1.8509, 41CrAlMo7; Ukrainian analog 38Cr2MoAl high quality) was chosen, which is subject to nitriding and is characterized by high hardness and wear resistance. The chemical composition of AISI A290C1M steel is presented in Table 1.

Chromium and aluminum form stable nitrides, which gives steel a very high hardness (up to HV 1100–1200). Chromium increases the hardness and strength of steel. Molybdenum is introduced into steel to eliminate tempering brittleness. At the same time, it increases hardenability.

### 2.2. Heat Treatment

**Figure 1:**
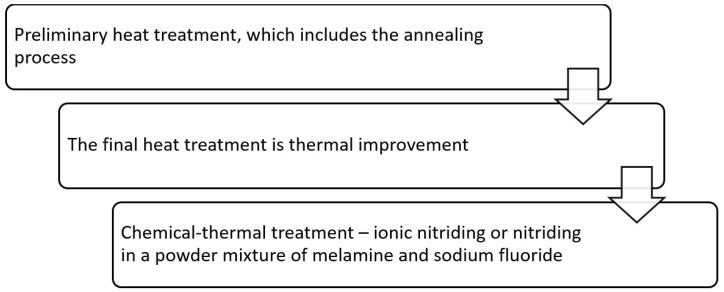
Scheme of the heat treatment process of AISI A290C1M steel.

Several thermal operations were required to obtain the necessary properties on the surface and in the core of the AISI A290C1M steel product (Figure 1).

Annealing is a preparatory operation in which castings, forgings, and rolled products are subjected to reduce hardness and strength and improve machinability by cutting steel. By crushing the grain, reducing internal stresses, and reducing structural heterogeneity, this heat treatment helps to increase plasticity. Full annealing aims to speed up the process, reduce strength after hot plastic deformation (forging), and prepare the structure for further heat treatment. This method is used to obtain a fine grain, uniform distribution of structural components, improve the machinability of low-carbon steel by cutting, eliminate the carbide grid, improve mechanical properties, and reduce the cold brittleness threshold. Annealing was carried out with heating to a temperature of 880–900 °C, the holding time was 1 h, and the cooling medium was with a furnace.

Compared to annealing, quenching with high tempering (thermal improvement) creates the best ratio between the strength of the steel and its plasticity. Quenching of steel AISI A290C1M was carried out with heating to 930–950 °C, the holding time was 15 min, and the cooling medium was oil. Standard mineral oil for tempering with increased antioxidant properties of the MZM-26 type was used, which is intended for tempering steel products made of low-alloyed and alloyed steels at working temperatures of 30–120 °C. The oil can be used both indoors and outdoors. It is made based on high-quality mineral basic components of deep cleaning and effective composition of additives, provides deep and high-quality hardening, including large-sized products of complex shape, has high chemical resistance, and does not enter a reaction with the hardened metal. During quenching, the necessary hard and strong structure, martensite is formed. The most important operation during quenching is cooling. At high cooling rates during quenching, internal stresses arise that can lead to distortion or cracking of the part. The causes of internal stresses are different temperatures across the cross-section of the product; they are called thermal. Phase voltages are still formed; choosing a suitable cooling medium is necessary to reduce this. Oil is used as a quenching medium for the hardening of small parts with a section of up to 5 mm made of carbon steel and large diameter parts made of alloy steel. For AISI A290C1M steel, oil is used as a cooling medium, as oil reduces defects from cracks [27,28].

A mandatory heat treatment operation after quenching is steel tempering to reduce brittleness and internal quenching stresses and obtain the required strength properties of steel. Tempering was carried out at a high temperature of 640–660 °C, which exceeds the maximum temperature of the subsequent nitriding, which ensures the hardness at which the steel can be processed by cutting. The cooling medium was air. The duration was 1 h. The structure after such processing is sorbitol.

Chemical-thermal treatment—nitriding, in our case ionic or nitriding in a powder mixture of melamine and sodium fluoride.

To conduct the experiment, samples of 2.0 × 3.0 × 1.5 cm were used, for which the holding time for the processes was chosen accordingly.

Annealing, quenching, and high tempering of the samples were carried out in a laboratory electric furnace FE—0.005/1100 (F—furnace; E—electric; 0.005—useful volume of the working chamber, m^3^; 1100—maximum heating temperature, °C). Technical characteristics: working space height—200 mm, width—300 mm, depth—380 mm.

#### 2.2.1. Ion Nitriding

**Figure 2:**
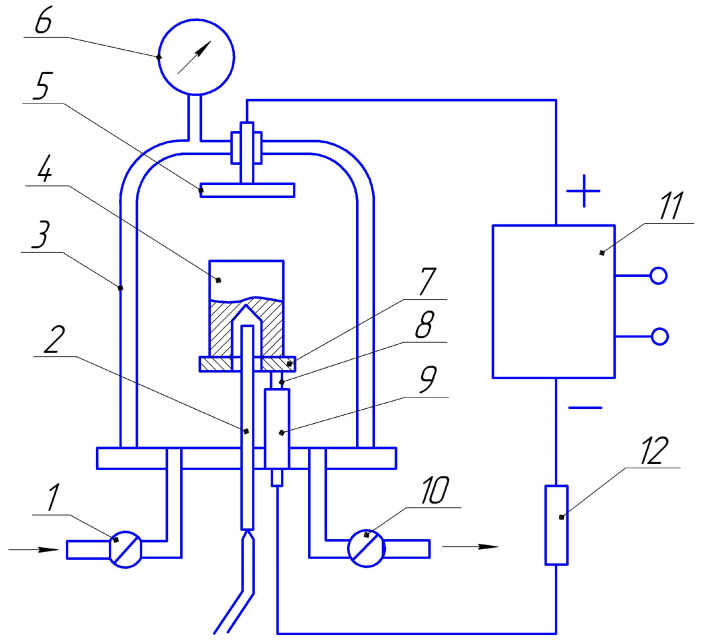
Scheme of the installation for heating and chemical-thermal treatment of metals in a glow discharge: 1, 10—valves for regulating the gas flow and pressure; 2—thermocouple for measuring the temperature of the processed part; 3—reaction chamber; 4—sample; 5—anode; 6—manometer for measuring gas pressure in the chamber; 7—sample stand; 8—cathode; 9—insulating tube; 11—voltage source; 12—limiting resistance.

Installations are used to heat metals and alloys in a glow discharge and chemical-thermal treatment. The principle of operation is presented in Figure 2. The processed part 4 is placed in the gas discharge reaction chamber 3. The reaction chamber must be sufficiently airtight and have a small influx of air from the atmosphere. The reaction chamber has a system of pumping out and supplying gas medium.

A vacuum rotary oil pump (fore vacuum pump) is enough to pump out air or gas. Valves 1 and 10 are used to regulate the flow rate and pressure of the gas medium. Manometer 6 measures the gas pressure in the chamber. Electrodes are introduced into the chamber through sealed insulators. One of the electrodes—the cathode—must apply a negative potential to the processed parts. In Figure 2, the cathode is shown as rod 8, which ends with a stand 7 under sample 4. The rod is insulated with a quartz or porcelain tube 9. The discharge anode is electrode 5, which is specially inserted on the camera’s metal body. The distance between the anode and the cathode is not critical and can vary widely. As a result, the smoldering glow evenly covers the surface of parts with a complex configuration, ensuring a uniform supply of energy to the surface of the heated part. The glowing discharge is fed with direct or pulsating current from a special voltage source 11, which contains a high-voltage rectifier with a voltage of up to 2000 V. To prevent the glow discharge from turning into an arc, the current is limited using resistance R.

**Figure 3:**
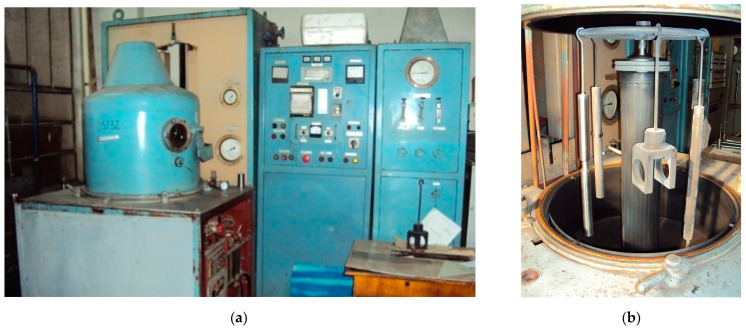
General view of the unit for ion nitriding type NGV-6.6/6-I1 (**a**) and location of parts in the chamber (**b**).

To carry out ion nitriding, the NGV-6.6/6-I1 electric furnace (NICMAS, Sumy, Ukraine) was used in the work, which is designed for nitrogen saturation of the surface layers of machine parts and tools (Figure 3a). The main components of the installation are a reaction chamber and a cabinet with a power supply device. This unit controls electronic equipment, a gas preparation unit, and a gas pumping system.

**Figure 4:**
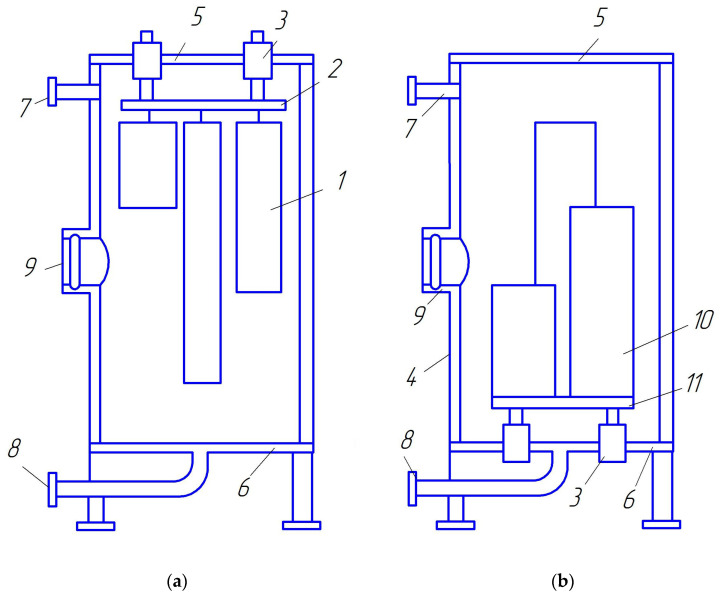
Schematic placement of parts in the ion nitriding installation chamber: 1—details in a suspended state; 2—ring for hanging parts; 3—current leads with hermetic insulators; 4—camera; 5—upper bottom; 6—lower bottom; 7—nozzle for gas injection; 8—nozzle for pumping out the gas; 9—inspection window; 10—parts installed on the plate; 11—plate-stand for parts. Upper placement of parts (**a**), lower placement of parts (**b**).

The parts can be placed in the chamber in a suspended state (Figure 3b and Figure 4a) or on the plate near the lower bottom (Figure 4b). The ring at the upper bottom, on which the parts are suspended, and the plate at the lower bottom are fixed on the bottoms through hermetic insulators. They are current leads of negative potential to the parts and, together with the parts, are the cathode of the discharge. The camera itself is used as an anode.

The technological process of ion nitriding consists of two main operations—cathodic sputtering and direct ion nitriding. The sequence of operations during the technological cycle was as follows. Parts subjected to nitriding were installed in the chamber and connected to the negative electrode. The chamber was sealed, the air was pumped out to a pressure of 1.4 × 10^2^ Pa, and the chamber was blown with working gas for 5–15 min at a pressure of 13 × 10^2^ Pa. Then the chamber was pumped down to a pressure of 26–52 Pa, a voltage was applied to the electrodes, and a glow discharge was excited. The temperature of ion nitriding was 550 °C, and the holding time was 7 h. After isothermal holding, the parts were cooled down to room temperature under a vacuum.

#### 2.2.2. Nitriding in a Powder Mixture of Melamine and Sodium Fluoride

Melamine (C_3_H_6_N_6_) is an organic base, a nitrogen-containing, colorless, crystalline chemical that is odorless and practically insoluble in cold water and most organic solvents. The content of atomic nitrogen in melamine is 66.67%, and the content of atomic carbon is 28.57%, so the proposed composition allows simultaneously saturating parts made of steel and titanium alloys with nitrogen and carbon. The melting point of melamine crystals is 354 °C. When heated above the melting temperature, melamine breaks down with the formation of nitrogen free radicals and melem (C_6_H_6_N_10_, gray amorphous substance); above 450 °C, the melem decomposes by releasing additional free nitrogen radicals. The total amount of nitrogen free radicals during nitriding using melamine is up to 70%, which subsequently forms the diffusion layers on parts made of steel and titanium alloys.

Sodium fluoride is added to the mixture in the amount of 5% to activate the nitriding process. It is an inorganic binary compound with the chemical formula NaF, a white crystalline substance. It is used as a component of compositions for cleaning metals, fluxes for welding, soldering, and remelting of metals, glass, enamels, ceramics, refractories, as a component of acid-resistant cement, heat-resistant materials, compositions for etching glass, solid electrolytes, etc. When the content of the activator is increased by more than 5%, the saturation activity does not increase, so it makes no sense to increase its amount. When the content of the activator is less than 3%, the nitride layer’s thickness decreases, reducing the products’ operational properties.

The amount of the mixture was chosen from the point of view of the required layer thickness, its hardness and depth distribution, and the quality of the diffusion layer. To study the influence of melamine on the speed of formation and properties of diffusion layers, a mixture was used in the ratio of components, wt. %: melamine (C_3_H_6_N_6_)—95, sodium fluoride (NaF)—5. When its content is increased above 5%, the saturation activity does not increase. When the activator content is less than 5%, the nitride layer’s thickness decreases, reducing the products’ operational properties. To conduct the experiment, the amount of the mixture was taken from the following ratio of 2.3 g/cm^2^ of the treated surface. Next, the experiment was carried out with the specified amount of powder mixture.

Traditionally, the gas nitriding temperature for AISI A290C1M steel is 520–550 °C. Given that the optimization indicators are the ratio of surface hardness and thickness of the nitride layer, the optimum nitriding temperature for steel is 550 °C.

To conduct the experiment, the samples were placed in a container filled with the selected mixture, after which the container was hermetically closed and placed in the furnace. Technological parameters of nitriding: process temperature—550 °C, holding time—5 h, cooling medium—air. To perform nitriding in a powder mixture of melamine and sodium fluoride, an FE—0.005/1100 furnace was used in the work.

### 2.3. Research Methods

Hardness measurements were carried out using the Rockwell method on the TK-2 device. Nitriding results were monitored by measuring surface microhardness on a PMT-3 device with a load of 50 g.

Microhardness tests on PMT-3 were conducted in the following way. The plate with a sample was installed on the table; the research object was placed under the microscope lens. After focusing the microscope on the sharpness of the image and inspecting the area intended for testing, a place was chosen to determine the microhardness. The sample was placed on a table mounted on a screw, which moved by turning the flywheel until the diamond pyramid collided with the surface of the sample. The sample was loaded by slowly (10–15 s) turning the handle of the indenter stop and held for 5 s, after which the stop handle was returned to its original position. When turning the table in the opposite direction as far as it will go, the specified research area returned to its previous position. From this, it followed that the indenter could be pressed precisely in the selected place. By turning the microscope head to the right as far as it will go, the microscope lens was connected to the print. The print was focused, and the size of the diagonals was measured. When counting, we used a microscope scale, one division of which is exactly 0.1 mm, and a microscrew, one division of which on the limbus corresponds to 0.001 mm. Using tabular data, the Vickers hardness (HV) was determined by the d value.

A Neofot-21 metallographic microscope (Carl Zeiss, Jena, Germany) was used to study the microstructure of the obtained samples.

When developing new materials, a fairly accurate experimental evaluation of the advantages of the material tested for friction in conditions close to real operating conditions is required. The AISI A290C1M steel studied in the work is used for the manufacture of nitriding parts: gears, rollers, rods, plungers, bushings, details of rocket and aircraft engines, etc., operating at temperatures up to 450 °C. According to the conditions of use of these parts, it is necessary to choose the most rational method of nitriding the surface of such details so that they have optimal strength and wear resistance when used in products.

**Figure 5:**
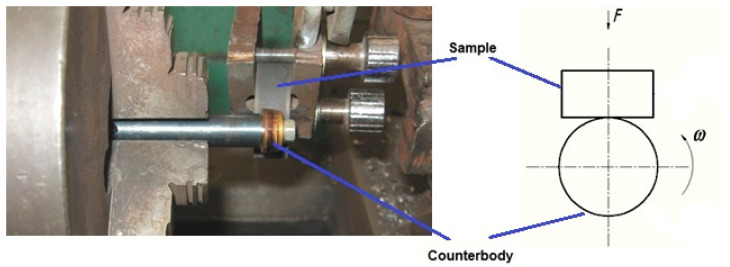
The process of sample wear on a lathe and screw-cutting machine and the scheme of wear resistance tests.

One main direction that helps solve the task is a correct and justified sequence of conducting laboratory tribometric tests supported by appropriate physical methods. The behavior of a real friction unit or a specific part of it in such experiments is simulated with the help of a laboratory-tested system, which simulates its behavior during operation to one degree or another. Wear tests were performed with friction without lubrication on a lathe and screw-cutting machine model 1A616P in a specially developed device in the laboratory of Sumy State University. The contact was implemented on the side surface of the sample according to the wear scheme presented in Figure 5.

To carry out tests on wear resistance, the samples were installed in the clamp of a special device, and the counterbody was fixed in the chuck of the lathe and screw-cutting machine. The samples were fixed so that their surface was parallel to the surface of the counterbody. Further processing of the results was performed depending on the area of surface wear.

The preparation of samples for wear resistance tests included several stages. First, by processing with the help of cleaning methods, it is necessary to remove all oil contamination. Then, all other impurities were removed with sandpaper of the smallest grain. At the end of the preparation, the samples are degreased with ethyl alcohol.

The main parameters of the test are indicated in Table 2.

Wear resistance tests were carried out by two methods. Firstly, the amount of wear was estimated by comparing the weight of the sample before and after the tests. Weighing was carried out on high-precision analytical balance BA-200. A series of studies consisting of three experiments was performed (three samples for each type of heat treatment for which a comparison was made). The deviation of the weight of the samples in each series of experiments was ±0.00015 g.

Secondly, a metallographic analysis of the wear pits was carried out, providing information about the wear’s nature. The amount of wear was determined by measuring the area of the wear zone.

## 3. Results

Thermal and chemical-thermal treatment modes were proposed for strengthening the surface of the AISI A290C1M steel sample (Table 3) and obtaining the necessary material characteristics, considering the acceleration and cost reduction of chemical-thermal treatment processes:

1. Annealing, quenching, high tempering, ion nitriding;
2. Annealing, quenching, high tempering, nitriding in a melamine and sodium fluoride mixture.

**Figure 6:**
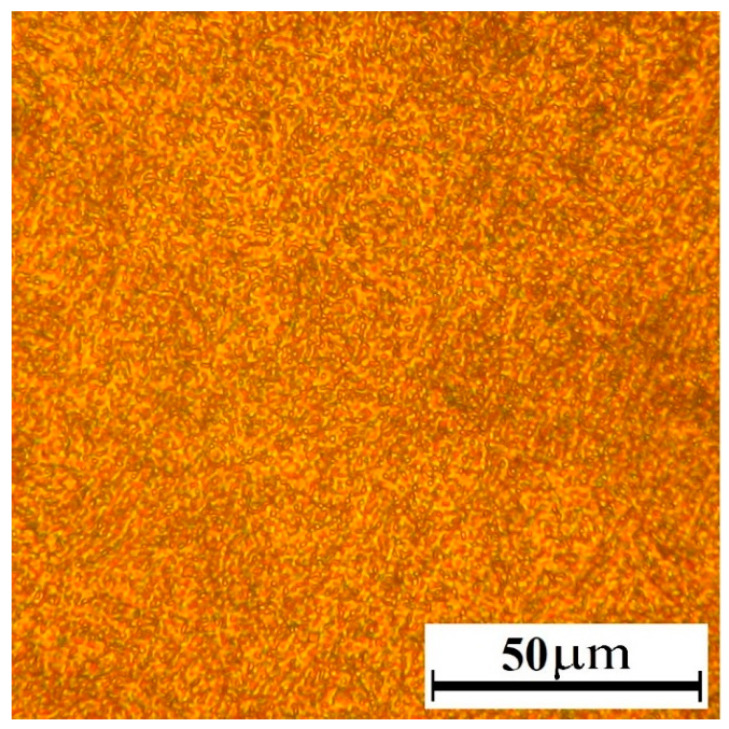
Structure of AISI A290C1M steel after high tempering.

After annealing, a structure consisting of ferrite and pearlite was obtained. The hardness was HB 208. The structure after quenching is martensite. The hardness was HRC 64–66. The structure after tempering and before nitriding was tempering sorbitol with a hardness of HRC 40–50 (Figure 6).

### 3.1. Results Obtained after Ion Nitriding

**Figure 7:**
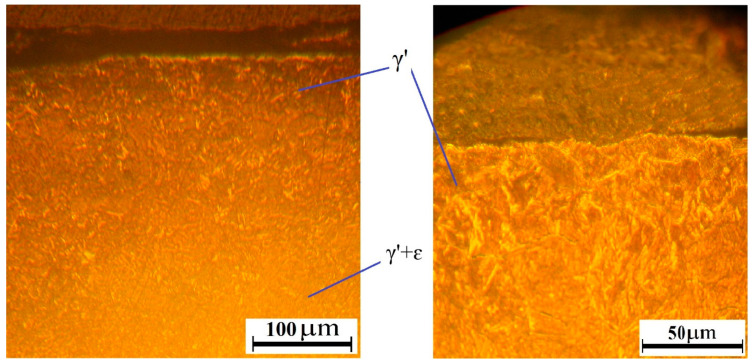
Structure of AISI A290C1M steel after ion nitriding.

The microstructure of AISI A290C1M steel after ion nitriding is presented in Figure 7. After carrying out the ion nitriding process, a hardness of HV 950–1000 was obtained, and the depth of the saturated nitride layer was 0.25–0.32 mm.

Analysis of the microstructure showed that the outer nitride layer has no cracks. As a result, a microstructure was obtained with the phase composition of the surface nitride layer consisting of the γ′-phase (a solid solution based on iron nitride with the composition Fe_4_N). The diffusion layer placed under the nitride layer included the release of carbonitrides (γ′ + ε) (ε–phase (hexagonal carbonitride Fe_2–3_(NS)), which form a grid. In the core, there was tempering sorbitol.

**Figure 8:**
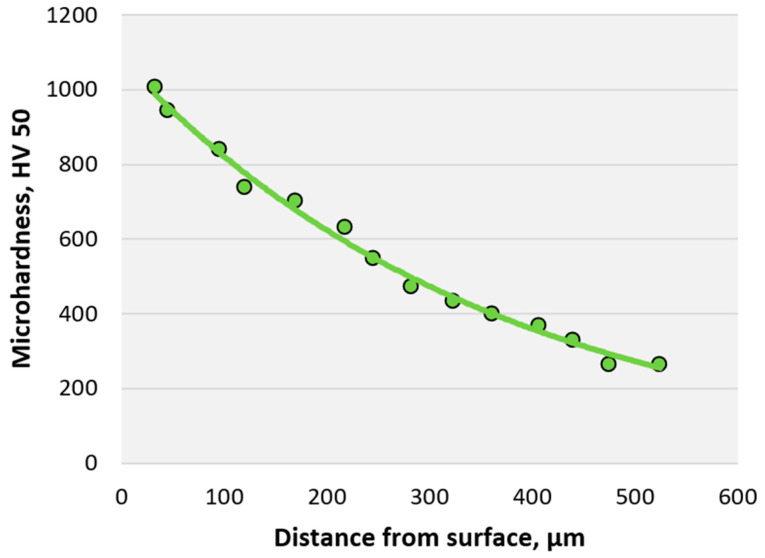
The graph of changes in the microhardness of AISI A290C1M steel after ion nitriding depends on the distance from the surface.

The change in microhardness after ion nitriding, depending on the distance from the surface, is shown in Figure 8.

Figure 8 clearly shows that as the thickness of the nitride layer changes, the microhardness of the sample decreases. It is due to a decrease in the nitrogen content in the steel. The thickness of the nitride layer is 0.25–0.32 mm.

### 3.2. Results Obtained after Nitriding in a Powder Mixture

**Figure 9:**
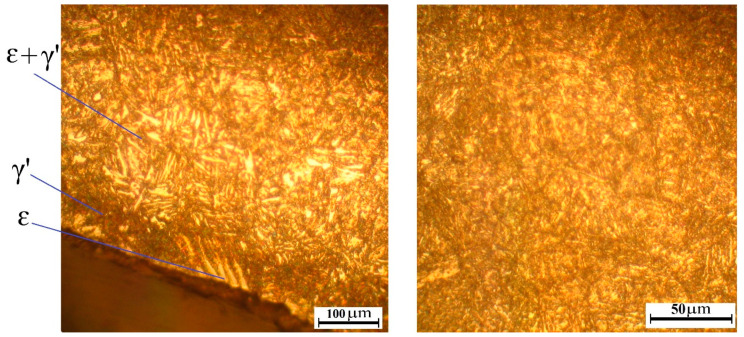
Structure of AISI A290C1M steel after nitriding in the mixture.

The microstructure of AISI A290C1M steel after nitriding in a melamine mixture is shown in Figure 9. The microstructure and phase composition of the diffusion layers formed during nitriding in a melamine mixture do not differ from those formed under the conditions of traditional gas nitriding: a thin surface layer consisting of ε (solid solution based on iron nitride Fe_4_N) and ε + γ′ (solid solution on based on iron nitride Fe_2–3_N) phases. At the same time, there are significant differences in the diffusion process. It is significantly accelerated, and the increase in the thickness of the nitride layer occurs mainly due to the internal nitriding zone.

**Figure 10:**
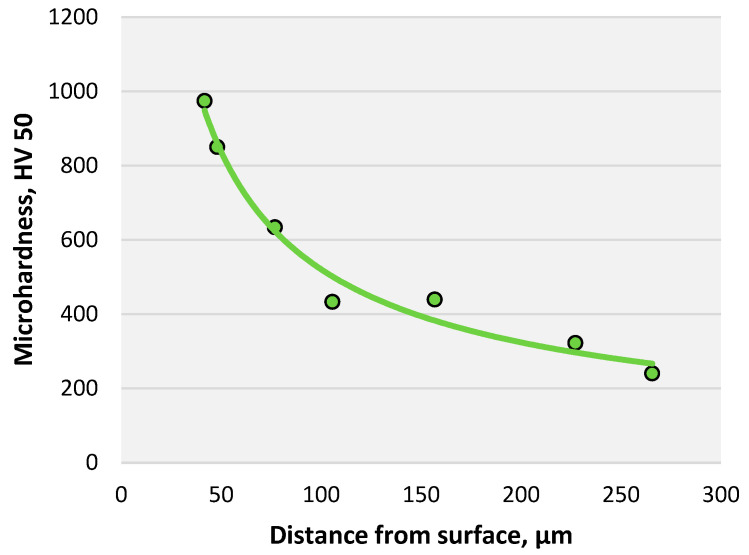
The graph of changes in the microhardness of AISI A290C1M steel after nitriding in the mixture depends on the distance from the surface.

The change in microhardness after nitriding in the mixture, depending on the distance from the surface, is shown in Figure 10. It can be seen from the graph that the microhardness decreases with the change in the thickness of the nitride layer. The thickness of the layer is about 0.2 mm.

As a result of the conducted experiment, it was found that using a mixture of melamine with sodium fluoride significantly accelerates the nitriding process. However, nitriding duration, set for 5 h, is ineffective since the required layer thickness was not obtained, but the obtained hardness satisfies the required indicators—HV 970. It means that the process needs to be improved.

### 3.3. Results of Wear Resistance Tests

Wear tests of AISI A290C1M steel were carried out after the different modes of thermal operations. The results of wear resistance tests are given in Table 4.

From the obtained data, the wear resistance of the samples subjected to thermal improvement and ion nitriding increased by 2.17 times, and those subjected to thermal improvement and nitriding in a powder mixture (95% melamine + 5% sodium fluoride) increased by 2.6 times. Steel is subject to the least wear after nitriding in a powder mixture.

## 4. Discussion

For AISI A290C1M steel, the following mode of ion nitriding was proposed: temperature—550 °C, holding time—7 h. After carrying out the process, the hardness HV 950−1000 and the depth of the saturated layer 0.25–0.32 mm were obtained. The use of this mode of ion nitriding allowed for the reduction of the duration of the technological cycle by 5–6 times compared to two-stage nitriding and to optimize the composition of the diffusion layer. It consisted of a nitride layer on the surface (γ′-phase) and had no cracks. The diffusion layer, placed under the nitride layer, included the allocation of carbonitrides, which form a grid. In the core, there was tempering sorbitol and carbides.

**Figure 11:**
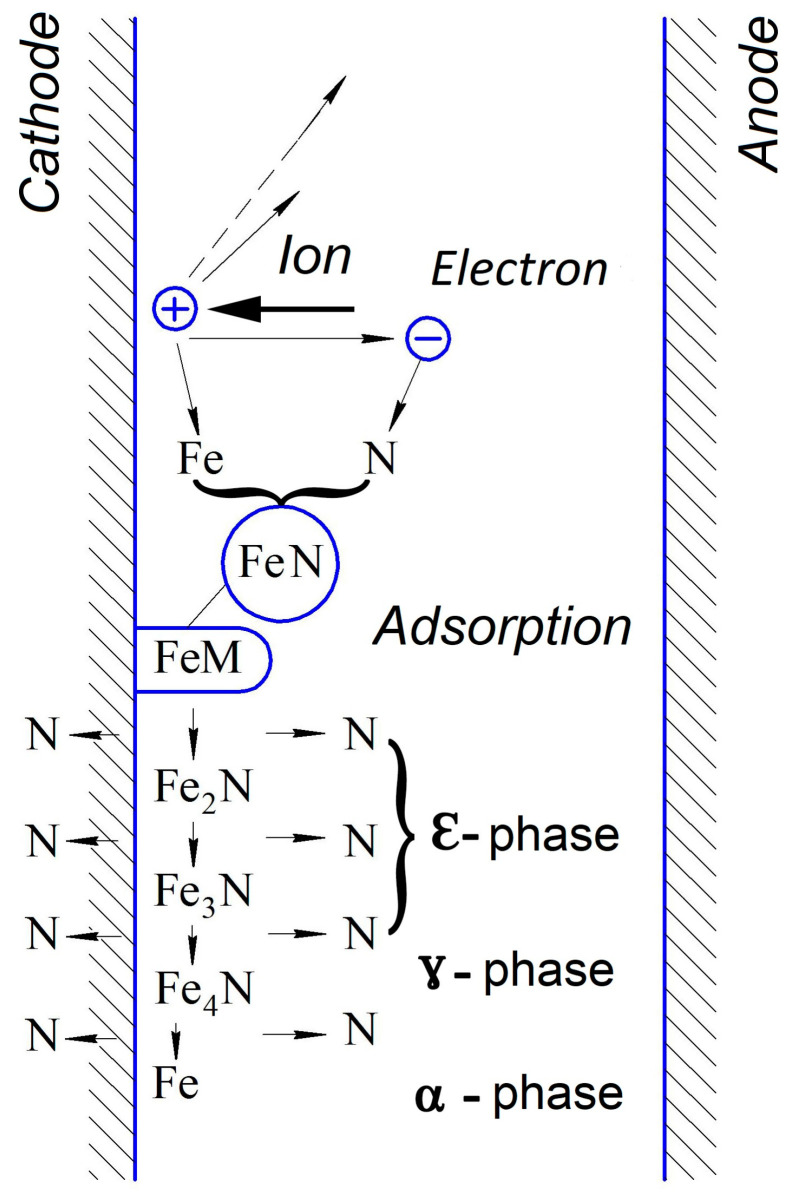
Mechanism of ionic nitriding.

As a result of the ionization of the gas medium, ionic nitriding can be carried out in ammonia, a mixture of nitrogen and hydrogen, and pure nitrogen. Atomic nitrogen ions are responsible for the nitriding process. As a result of the cathodic sputtering of iron near the part’s surface, FeN iron nitride molecules are formed in a gas environment (Figure 11).

As a result of reverse cathodic sputtering, FeN molecules settle on the saturated steel surface and release nitrogen atoms that diffuse deep into the metal to form a solid solution in iron. The processes of cathodic sputtering in iron and reverse cathodic deposition of nitride occur simultaneously during nitriding and can be regulated by the pressure of the gas medium. The gas pressure during nitriding, depending on the required structure of the layer, is set in the range of 10–1000 Pa. At low pressure, cathodic sputtering prevails, and the amount of nitrogen in the gas medium is small. Under such conditions, a diffuse layer of a solid solution of nitrogen in iron develops, and the nitride zone of the nitride layer does not form. In the case of increased pressure, the reverse cathodic sputtering is so noticeable that a zone of iron nitrides is formed immediately after the start of the process. The ratio between sputtering and reverse cathodic deposition depends on the discharge voltage. As a result of the selection of the specified process parameters, it is possible to obtain nitride layers with a diverse structure and composition, which will satisfy the most diverse requirements regarding the plasticity of the nitride material, surface hardness, and corrosion resistance.

**Figure 12:**
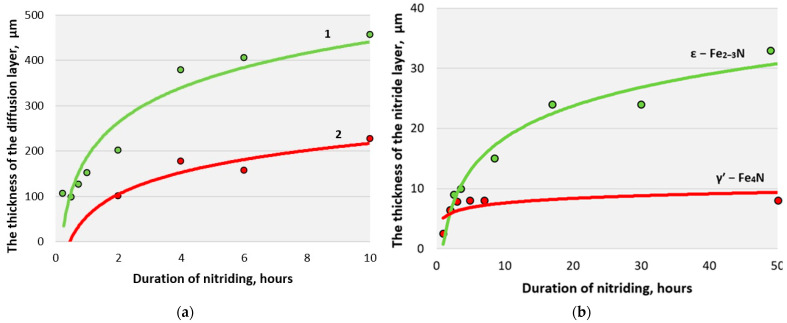
Effect of nitriding duration on the thickness of diffusion (**a**) and nitride layers (**b**) of AISI A290C1M steel: 1—ion nitriding; 2—furnace nitriding.

A significant reduction in the total time of the technological cycle (by 3–5 times) is achieved due to a reduction in the time of heating and cooling of the cage and a reduction in holding time. The thickness, kinetics of formation, and structure of the diffusion layer depend on the temperature, duration of the process, rarefaction, and composition of nitrogen-containing atmospheres (Figure 12).

The phase composition of the outer nitride layer obtained in a glow discharge differs from the phase composition of the nitride layer obtained by conventional gas nitriding. The nitride layer consists of γ′– or ε–phase, while during gas nitriding, a nitride layer consisting of a mixture of γ′– and ε–phases is obtained. The nitride layer obtained by ionic nitriding does not have cracks and is characterized by increased plasticity. The diffusion layer, located under the nitride, obtained during ion nitriding, contains fewer carbonitrides that form a grid or do not contain them, increasing the nitride layer’s plasticity. The layer with a minimal nitride zone or no nitride zone at all, formed under strong cathodic sputtering conditions and low nitrogen content in the gas medium, has the greatest plasticity.

According to the research results, it was established that ion nitriding, compared to traditional furnace nitriding, allows for achieving high saturation rates, possibilities of adjusting the composition of the diffusion layer, increased plasticity, and reduced distortion of nitride parts, reduction of the overall time of the process. This process is economical and environmentally friendly. Using ion nitriding shortens the technological cycle’s duration, optimizes the diffusion layer’s composition, provides a technologically simple process automation scheme, and improves the quality of nitride coatings.

Experimental studies were also proposed and conducted on AISI A290C1M steel using a saturating medium based on nanodispersed melamine powder (particle size 20–30 nm), which accelerates the nitriding process of steel products, compared to traditional nitriding. Melamine (C_3_N_6_H_6_) is an organic base and trimer of cyanamide with a 1,3,5-triazine structure. Like cyanamide, 66% (by mass) consists of nitrogen (atomic nitrogen content is 66.67%, and atomic carbon is 28.57%). Therefore, it allows parts made of steel and titanium alloys to be simultaneously saturated with nitrogen and carbon.

The technical result is ensured by the powder mixture containing sodium fluoride at the following ratio of components, wt. %: melamine—97–95, sodium fluoride—3–5. The proposed composition allows nitriding to be carried out in a closed atmosphere in the furnace without complex special equipment, without protective atmospheres during exposures of different durations, and without using gases harmful to human health, such as ammonia. Sodium fluoride is added to the mixture in the amount of 3–5% to activate the nitriding process. When the activator’s content is increased by more than 5%, the saturation activity does not increase, so it makes no sense to increase its amount. When the content of the activator is less than 3%, the nitride layer’s thickness decreases, reducing the products’ operational properties.

Due to the use of the proposed composition of the powder mixture during nitriding, it is possible to simplify the technological process significantly. There is no need to prepare complex mixtures, no coating with subsequent drying on the surface of the parts, and no need for additional expensive equipment, which, in turn, significantly speeds up and simplifies the nitriding process, which is significantly more energy-saving and economical.

The process parameters were chosen: temperature 550 °C, holding time—5 h, amount of melamine—2.3 g/cm^2^ of the treated surface. As a result of the conducted experiment, it was found that using a mixture of melamine with sodium fluoride significantly accelerates the nitriding process. A significant increase in the nitriding speed is due to a much higher amount of atomic nitrogen when using melamine instead of ammonia and the practically simultaneous disintegration of nanodispersed particles upon reaching the nitriding temperature. The apparent increase in the growth rate of the nitride layer is because during short-term nitriding (5 h), the primary role is played by the acceleration of nitrogen diffusion due to a greater number of defects in the crystal structure, and its inhibition due to interaction with carbon and alloying elements has not yet occurred. However, nitriding duration, set for 5 h, is ineffective since the required layer thickness was not obtained, but the obtained hardness satisfies the required indicators—HV 970. It means that the process needs to be improved.

The obtained wear resistance test data established that the wear resistance of the samples subjected to thermal improvement and ion nitriding increased by 2.17 times, and those subjected to thermal improvement and nitriding in a powder mixture increased by 2.6 times. Steel after nitriding in a powder mixture is subject to the least wear. These data are confirmed by the results of the studies [29,30].

Analysis of modern methods of surface strengthening of parts by chemical and thermal treatment showed that, despite the use of several new impregnation media and equipment, the problem of accelerating processes is still not solved. Therefore, developing technologies that would significantly reduce processing time, simplification, and cost reduction without deterioration of product properties remains an urgent issue.

## 5. Conclusions

During the execution of the work, the most rational methods of carrying out the process of ionic nitriding or nitriding in a powder mixture were chosen and substantiated; the advantages of these processes compared to gas nitriding were indicated.

For AISI A290C1M steel, the following mode of ion nitriding was proposed: temperature—550 °C, holding time—7 h. The hardness HV 950–1000 and the depth of the saturated layer 0.25–0.32 mm were obtained. It made it possible to reduce the duration of the technological cycle by 5–6 times compared to two-stage nitriding to optimize the composition of the diffusion layer.

The result of an experiment conducted on AISI A290C1M steel using a saturating medium based on nanodispersed melamine powder when applying the process parameters (temperature—550 °C, exposure time—5 h, amount of melamine—2.3 g/cm^2^) of the treated surface revealed that the use of the mixture of melamine with sodium fluoride significantly accelerates the nitriding process. However, the nitriding duration set at 5 h is ineffective because the required layer thickness was not obtained, but the obtained hardness satisfies the required indicator—HV 970. This means that the process needs to be improved.

The use of a mixture of 95% melamine and 5% sodium fluoride during nitriding makes it possible to simplify the technological process significantly: not to prepare complex mixtures, not to apply smears with subsequent drying on the surface of the parts, and not to require additional expensive equipment, which in turn significantly speeds up and simplifies the nitriding process.

It was established that the wear resistance of the samples that were subjected to quenching, high tempering, and ion nitriding increased by 2.17 times, and those that were subjected to quenching, high tempering, and nitriding in a powder mixture (95% melamine + 5% sodium fluoride) increased by 2.6 times. Steel after nitriding in a powder mixture is subject to the least wear.

## Figures and Tables

**Table 1:** The chemical composition of AISI A290C1M steel, %. wt.

C	Si	Mn	Ni	Cr	Mo	Al	Cu	S	P
0.35−0.42	0.20−0.45	0.30−0.60	up to 0.30	1.35−1.65	0.15−0.25	0.7−1.1	up to 0.30	up to 0.025	

**Table 2:** Parameters of wear resistance tests.

Parameter	Value
Counterbody material	Ti-Ta-W-Co alloy (with vacuum sputtering of the surface with titanium nitride)
Counterbody diameter, mm	15
Counterbody rotation frequency, rpm	355
Counterbody hardness, HV	1800
Load, MPa (considering the system of levers)	0.1
Test time for each of the samples, s	3600

**Table 3:** Modes of thermal and chemical-thermal treatment for AISI A290C1M steel.

Mode №	Type of Processing	Temperature Mode, °C	Holding Time, h	Cooling Medium
1	Annealing	900	1	with furnace
2	Quenching	950	0.25	oil
3	High tempering	660	1	air
4	Ion nitriding	550	7	with furnace
5	Nitriding in a mixture	550	5	air

**Table 4:** Results of wear resistance tests.

Indicator	Modes of Thermal Operations		
	1	2	3
Hardness	HB 208	HV 1000	HV 970
Weight of the sample before testing, g	70.22896	70.88233	69.98961
Weight of the sample after testing, g	69.6625	70.62129	69.77175
Loss of weight of the sample during the test, g	0.56646	0.26104	0.21786
Wear area, mm^2^	13	6	5
Coefficient of wear resistance *	1.00	2.17	2.60

* Accept the coefficient of wear resistance of AISI A290C1M steel after annealing as 1.00.

## Data Availability

The data presented in this study are available on request from the corresponding author.
